# Exploring the current status of pharmacist prescribing in Middle Eastern Arab countries: a scoping review

**DOI:** 10.1007/s11096-026-02108-0

**Published:** 2026-03-17

**Authors:** Rafah H. Alajmi, Heather E. Barry, Haya M. Almalag, Carmel M. Hughes

**Affiliations:** 1 Primary Care Research Group, School of Pharmacy, Medical Biology Centre, Queen’s University Belfast, 97 Lisburn Road, Belfast, BT9 7BL Northern Ireland, UK; 2 Department of Clinical Pharmacy, College of Pharmacy, King Saud University, Riyadh, Saudi Arabia

**Keywords:** Middle East, Non-medical prescribing, Pharmacist, Scoping review

## Abstract

**Introduction:**

The practice of prescribing by healthcare professionals other than physicians is called non-medical prescribing. Pharmacist prescribing (PP) is established in various countries and is expanding globally. In Middle Eastern Arab countries, there are significant developments in pharmacy education and practice with an expansion in the scope of practice; however, little is known about PP in these nations.

**Aim:**

This scoping review aimed to explore the current status of PP in Middle Eastern Arab countries. The objectives were to identify countries reporting on proposed or developing PP initiatives and to describe pre-implementation planning, including reported qualifications, models of prescribing, and contributing barriers and facilitators.

**Method:**

A search was conducted for published studies using nine databases from inception to October 2024. Full texts of papers focusing on PP, conducted in Middle Eastern Arab countries, and written in English and Arabic were obtained. All eligible papers were uploaded into Covidence. Duplicate papers were removed, and titles and abstracts were independently screened by two reviewers. Full-text screening was conducted by two reviewers. Data were extracted into a charting table with the following characteristics: author, publication year, aim, study design, setting, country, model of prescribing, qualifications, facilitators, barriers, and study recommendations. Data were synthesised narratively in line with the review objectives.

**Results:**

In total, 202 papers were identified and following screening, 16 papers were included in this review. Most studies (n = 9) originated from Saudi Arabia, with others from Qatar (n = 5), Jordan (n = 1), and the United Arab Emirates (n = 1). The included studies were published between 2019 and 2024 in English. Most studies were conducted in hospital settings (n = 12), particularly within specialised clinics describing two prescribing models: collaborative and independent. The findings reflect inconsistency in reporting the qualifications required for prescribing. Facilitators were categorised into regulation, education/training, professional competence, interprofessional collaboration, infrastructure, awareness, and international collaboration. Barriers encompassed regulatory gaps, organisational deficiencies, professional limitations, interprofessional resistance, resource constraints, and limited evidence.

**Conclusion:**

This review provides an overview of the current landscape of pharmacist prescribing in Middle Eastern Arab countries, key facilitators, and barriers. While the evidence base remains limited, findings suggest growing interest and early developments.

**Supplementary Information:**

The online version contains supplementary material available at 10.1007/s11096-026-02108-0.

## Impact statements

- This review mapped the current landscape of PP in Middle Eastern Arab countries, identifying key facilitators and barriers to successful implementation.
- Addressing the identified facilitators and barriers is crucial to fully integrating PP into healthcare systems.
- Policy development, training programmes, legislative action, and future research could assist the integration of PP into healthcare systems in this region.

## Introduction

The scope of the pharmacy profession has been expanding in Middle Eastern Arab countries, with significant developments in pharmacy education and practice. This expansion has had a positive impact in various practice areas, including the extension of the pharmacist's scope of practice to clinically integrated practice [1]. Moreover, pharmacy education is undergoing a transition in many countries in this region [1, 2]. There has been a growing recognition of the need to enhance pharmacist involvement in patient-centred services [1, 3]. Several studies have highlighted the positive impact of pharmaceutical care services provided by pharmacists [1, 4, 5]. Overall, the evolving role of pharmacists is aimed at enhancing the quality of healthcare and ensuring better patient outcomes [1–3].

Middle Eastern Arab countries include the following 12 countries: Bahrain, Iraq, Jordan, Kuwait, Lebanon, Oman, Palestine, Qatar, Saudi Arabia, Syria, the United Arab Emirates (UAE), and Yemen [6]. These countries have achieved significant advances in their health systems, pharmacy practice and education over the past decades [7–9] and are currently experiencing immense population growth and improvements in key health indicators [10]. They are classified by gross national income into four categories: low-income (Syria and Yemen), lower-middle-income (Palestine), upper-middle-income (Iraq, Jordan, and Lebanon), and high-income (Bahrain, Kuwait, Oman, Qatar, Saudi Arabia, and the UAE) [11]. The physician-to-population ratio ranges widely from 0.3 per 1000 population in Yemen to 2.9 per 1000 in the UAE [12]. Moreover, these countries have different regulatory frameworks, education and training programmes for health care professionals [7]. As the population continues to grow and age, the need for accessible and efficient healthcare services grows. Such a need is of particular importance in countries with an increasing burden of chronic diseases such as diabetes and hypertension [7].

The adoption of pharmacist prescribing (PP) is expanding globally [13–17]. The prescribing practice of healthcare professionals other than physicians is known as non-medical prescribing. Non-medical prescribers include pharmacists who have attained an advanced qualification in prescribing and are registrants of their professional regulatory bodies [18]. Several countries have implemented PP, such as the United Kingdom (UK), Canada, the United States, Australia, and New Zealand [18–22]. PP provides valuable benefits for patients and healthcare systems, including better patient care and improved patient outcomes [23–25]. Two common models of PP have been identified in the literature: independent and collaborative [13, 16, 18, 19, 26]. In the independent prescribing model, the pharmacist is solely responsible for assessing the patients and making the decision to prescribe. This includes assessment, initiation of medication, and follow-up. Therefore, pharmacist independent prescribers are completely responsible for patient outcomes. In collaborative prescribing, there is a cooperative partnership between pharmacist prescribers and a physician, regulated by a Collaborative Practice Agreement (CPA) [13, 20, 26, 27]. Under a CPA, the physician is responsible for the diagnosis and the pharmacist is authorised to perform prescribing actions such as initiating, monitoring, continuing or discontinuing medications. The physician and pharmacist are both responsible for patient outcomes [13, 20, 26, 27]. The implementation regulations vary from country to country. For example, in the UK, pharmacist independent prescribers can prescribe medications within their scope of competence [14, 18, 21, 22].

PP represents a novel area of practice in Middle Eastern Arab countries [6]. There is growing interest in implementing PP in some countries in this region, with varying levels of progress across various countries. However, research on this topic remains limited [28–30]. Therefore, this scoping review is essential for gaining a deeper understanding, thereby contributing to the existing knowledge on PP in this region.

### Aim

This review aimed to map the current landscape of PP readiness across the 12 countries of the Middle Eastern Arab region [6]. The specific objectives were to identify countries reporting on the development or proposal of PP initiatives and describe pre-implementation planning, including the models of prescribing being followed or proposed, the reported qualifications to gain the authority to prescribe, and the barriers and facilitators that affect its implementation.

## Method

The scoping review was guided by the methodological framework proposed by Arksey and O’Malley [31]. The recommendations in the Preferred Reporting Items for Systematic Reviews and Meta-Analyses Extension for Scoping Reviews (PRISMA-ScR) checklist [32] were followed. The protocol was registered and made publicly available via Figshare.com (10.6084/m9.figshare.26397019.v1). This review adopted the Joanna Briggs Institute (JBI) approach using population, concept, and context (PCC) as a framework around which the eligibility criteria were developed [33], as shown in Table 1.

**Table 1:** Inclusion criteria

PCC framework	Inclusion criteria
Population (P)	All studies in peer-reviewed and grey literature focusing on PP
Concept (C)	Studies focusing on PP
Context (C)	Studies conducted in Middle Eastern Arab countries [6]
Language	English and Arabic

### Search strategy

A search was conducted for published English or Arabic language studies, as well as relevant grey literature, using nine electronic databases: Medline, Embase, Scopus, Cochrane Library, Cumulative Index to Nursing and Allied Health (CINAHL), Google Scholar, ProQuest, OpenGrey, and ProQuest Dissertations. Searches were conducted from inception to October 2024. Grey literature was defined as any research that has not been published through peer-reviewed journals, including policy documents, dissertations, and reports from regulatory bodies. Finally, a manual search of the references of included studies was conducted. A detailed search strategy is presented in Appendix 1.

### Study selection

**Fig. 1:**
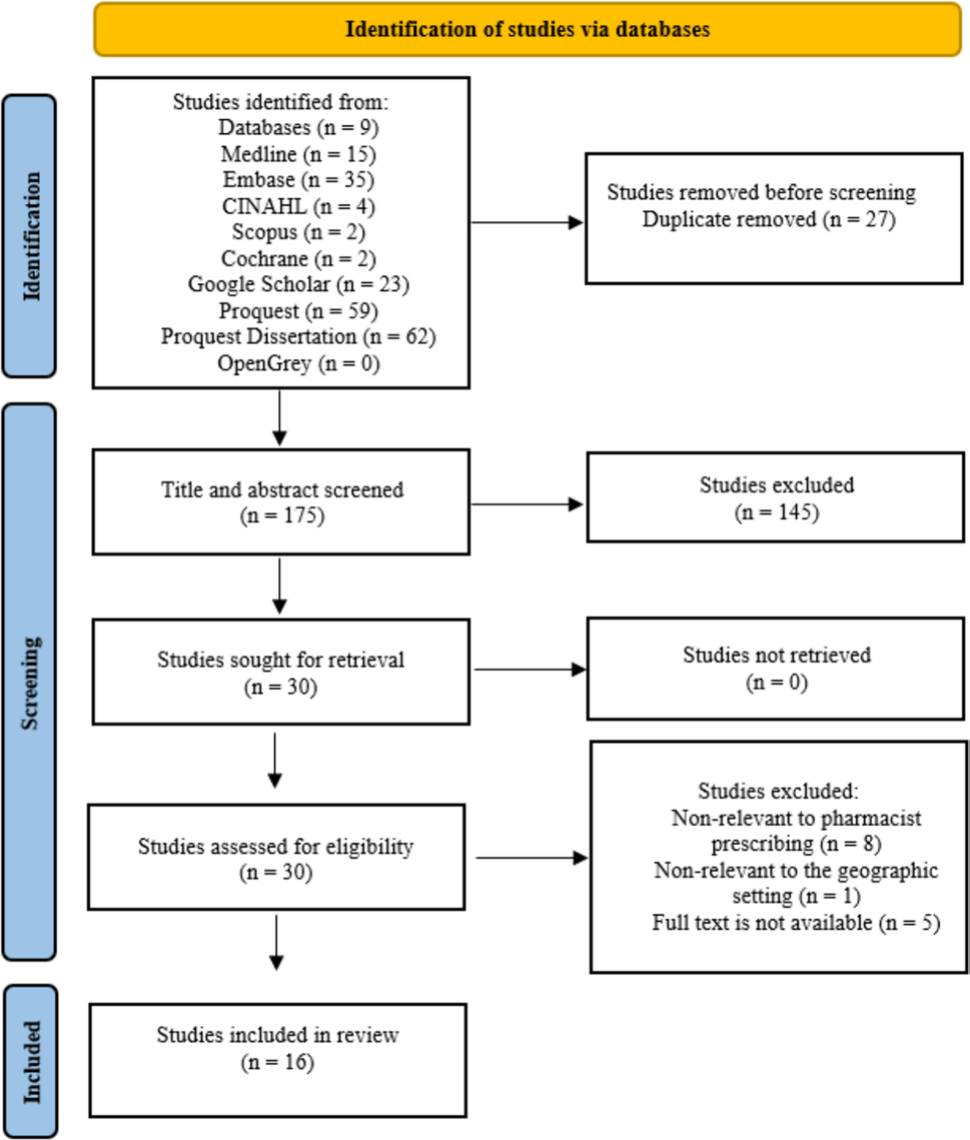
PRISMA diagram reporting the search and screening processes [32]

After uploading all identified papers into Covidence, duplicates were detected and removed. Next, two reviewers (RHA and HMA) independently screened the titles and abstracts of the remaining papers against the inclusion criteria in Table 1. Studies that did not meet the criteria at this stage were excluded. Finally, the full texts of all remaining papers were retrieved and independently assessed for eligibility by two reviewers (RHA and CMH). Where there was any uncertainty regarding the eligibility of some papers, guidance was sought and resolved by a third reviewer (HEB). The numbers of studies identified and selected are presented in Fig. 1, following the PRISMA-ScR guidelines.

### Data synthesis

Data were extracted into a charting table, which was piloted by extracting data from three studies. This process was verified for accuracy by all authors. The data were charted to provide an overview of lead author(s), publication year, study aim, study design, study setting, country, identified model of prescribing, reported qualifications for prescribing, reported facilitators, identified barriers, and study recommendations, presented in Appendix 2. A descriptive and narrative synthesis was used to summarise the data. Studies were grouped by country, model of prescribing, reported qualifications, clinical area, facilitators, and barriers, as described in the Results section.

## Results

The initial search yielded 202 papers. After removing duplicates (n = 27), 175 records were screened by title and abstract, of which 145 were excluded for not being related to the topic. Of the remaining 30 records that were fully reviewed, 14 papers were excluded, with eight not relevant to PP, one not relevant to the geographic setting, and five lacking full-text availability. The remaining 16 studies were eligible for inclusion as illustrated in a PRISMA flow diagram (Fig. 1). All included papers were published between 2019 and 2024 and were written in English. No eligible papers in Arabic were identified.

### Characteristics of the studies

Over half of the studies (n = 9) were conducted in Saudi Arabia [29, 34–41]. The other studies originated from Qatar (n = 5) [42–46], Jordan (n = 1) [47] and the UAE (n = 1) [30]. The majority of studies (n = 11) were conducted in hospital settings [29, 34, 35, 43, 47] and specialised clinics, including those for diabetes [37–39], anticoagulation [40, 41], and multiple myeloma [46]. Only one study was conducted in an academic institution [42] and one in a community pharmacy [30]. Three additional studies were grouped under the category "Other." These included a descriptive study detailing the pre-implementation and planning of a PP initiative for hospital settings and pharmacist-led clinics [36] and two broader investigations involving major healthcare institutions, as well as academic institutions and organisations engaged in pharmacy practice and regulation [44, 45]. The studies included employed various study designs. Characteristics of the included studies are shown in Table 2. The complete findings are presented in Appendix 2.

**Table 2:** Characteristics of included studies

Variable	Number of studies
*Study country*	
Saudi Arabia Qatar Jordan United Arab Emirates	9 5 1 1
*Study settings*	
Hospital Pharmacist-led diabetes clinic Pharmacist-led anticoagulation clinic Multiple myeloma clinic Academic institution Community pharmacy Other: Multiple healthcare institutions	5 3 2 1 1 1 3
*Study design*	
Cross-sectional Cohort Descriptive Observational Simulation study Curriculum mapping Qualitative Consensus-based Mixed method Non-interventional retrospective	4 3 2 1 1 1 1 1 1 1
Total	16

### Models of prescribing

The scoping review identified two models of PP: collaborative prescribing and independent prescribing. Collaborative prescribing, through a CPA, emerged as the most frequently reported model [29, 36–42, 46]. In contrast, independent prescribing was reported exclusively in the context of writing a Total Parenteral Nutrition (TPN) order [34–36]. While most PP practice in the region is hospital-based, this review found evidence from a community pharmacy setting where independent prescribing was explored as a potential future role based on community pharmacists’ perceptions and willingness to adopt the practice, suggesting a possible expansion beyond hospital settings for minor ailments management [30].

### Reported qualifications

While the included studies confirm the pre-implementation of PP, the pathways to achieving prescribing competency are not extensively addressed. Half of the studies did not specify any educational or training requirements. The other half reported qualifications and experience, including a Doctor of Pharmacy (PharmD) degree, a Master of Clinical Pharmacy, board certification, and residency training [29, 37, 47]. Additional qualifications were noted, such as certification in diabetes management [37, 39]. Additionally, some studies specified that the pathway to prescribing required pharmacists to be recognised as trained, certified clinical pharmacists and subsequently obtain clinical privileges. This involved a formal review process where they had to prove their competence to be granted a prescribing authority [38, 40, 41, 44].

### Facilitators of pharmacist prescribing

The analysis identified a range of facilitators, which were categorised into seven categories: regulation, education/training, professional competence, interprofessional collaboration, infrastructure, awareness, and international collaboration [29, 30, 36–40, 42, 43, 45–47]. Regulatory facilitators included the presence of institutional legislation that establishes CPAs with defined roles and responsibilities of pharmacist prescribers, and the alignment of pharmacist roles with national health strategies [29, 37–40, 45]. Identified facilitators included the qualifications of pharmacists, specialised education, such as PharmD and residency programmes, curriculum alignment with recognised prescribing competencies, and the provision of specialised training [30, 36, 38, 40, 42, 45]. For instance, the curriculum in Qatar University was reported to cover most of the prescribing competencies outlined in the Australian National Prescribing Services Prescribing Competencies Framework [42]. Additional identified facilitators were adherence to evidence-based guidelines and the application of clinical knowledge [36, 39, 40, 45, 47]. Interprofessional collaboration emerged as an important facilitator, evidenced by the support from physicians and multidisciplinary team collaboration [35, 36, 38, 43]. Moreover, the availability of clinic infrastructure and access to electronic records were critical [30, 38–40, 43]. Finally, efforts to enhance awareness of pharmacists' qualifications, along with establishing international collaborations with countries that have established PP, were also reported as facilitators [45]

### Barriers to pharmacist prescribing

Several barriers were identified and categorised into regulatory gaps, organisational deficiencies, professional and practice-related limitations, interprofessional resistance, infrastructure and resource constraints, and limited evidence [29, 30, 36, 38–40, 42, 43, 45–47]. Regulatory barriers included a lack of national legislation and limited regulations [29, 36, 42, 43]. Organisational deficiencies comprised the absence of administrative planning, a lack of organisation [36, 40], and the absence of defined roles for pharmacist prescribers [45]. Moreover, there is a lack of standardised definitions of PP models. Professional limitations involved insufficient training [29], existing gaps in the curricula [42], a lack of self-confidence among some pharmacists regarding their readiness to undertake a prescribing role [45], and a lack of dedicated time for prescribing due to competing workload [29]. Furthermore, a significant barrier identified was interprofessional resistance [30, 38, 43, 45, 47] and a concern regarding physician acceptance, with 35.9% of pharmacists facing resistance from physicians during their prescribing practice [29]. Moreover, qualitative findings provided deeper insights, revealing that physician resistance is often rooted in a fear of disrupting the established hierarchy [45]. Further study clarifies that this resistance arises when pharmacists are perceived to be taking on the role of diagnosis, which physicians consider a core part of their professional identity [43]. Furthermore, a study identified patient and public acceptance as a barrier to independent prescribing in community pharmacies, as it is uncommon for patients to receive prescribed medications from a pharmacist rather than a physician [30]. Limitations related to resources were also identified, such as inadequate infrastructure, staffing, and financial support [30], difficulties accessing patient information [42], and a reluctance to establish pharmacist-led clinics [39]. Finally, the scarcity of local data that demonstrate the effectiveness of PP [39], and the absence of patient satisfaction and cost-effectiveness evaluations [46] were reported as barriers.

## Discussion

This scoping review provides an overview of PP in Middle Eastern Arab countries, revealing an emerging but under-developed area of practice. The geographical distribution of the included studies revealed a significant concentration, with over half originating from Saudi Arabia [29, 34–41], followed by Qatar [42–46]. The remaining studies were from Jordan [47] and the UAE [30]. Consequently, only four of the 12 countries within the scope of this review were represented in the published literature on PP. This geographical concentration is likely attributed to the financial resources available in these wealthier nations, a pattern consistent with findings from other reviews of pharmacy practice research across the region [8]. Saudi Arabia has produced a growing number of studies that are likely influenced by its national Vision 2030 [48]. Launched in 2016, this strategy is a national framework designed to advance development across various sectors, including healthcare, and focuses on improving access to healthcare [48]. The methodological approaches employed by the included studies were diverse. The prevalence of cross-sectional and descriptive studies suggests an initial phase of research. This aligns with the fact that all included studies were published relatively recently (between 2019 and 2024), reflecting a growing but still emerging body of evidence on PP and the early stages of implementation in these countries.

There is a lack of standardised definitions of PP models in the region. However, this review identified two models of prescribing: independent and collaborative. Within the scope of this review, independent prescribing was identified in the context of TPN order-writing [34–36]. In this specialised, patient-specific area, order writing is equivalent to prescribing, which is consistent with surveys showing that pharmacists play a significant role in order-writing, either independently or as part of a nutrition support team [49, 50]. This also highlights a specific area within critical care where pharmacists have demonstrated a significant level of autonomy and specialised expertise, representing a strategic initial step for future expansion of their prescribing roles. Nevertheless, this exclusive context contrasts with the broader scope of independent prescribing roles observed internationally, where pharmacists manage a wider array of conditions and medications [13, 14, 19, 51, 52]. In addition, the review identified collaborative prescribing models in which pharmacists contributed as integral members of multidisciplinary teams. Similar collaborative arrangements have been reported internationally, where pharmacists manage defined areas of pharmacotherapy [13, 14].

Pharmacists are consistently required to practise within their area of competence, with a notable focus on diabetes, followed by anticoagulation and oncology clinics [29, 34, 35, 37–41, 43, 45, 46]. This observation suggests that PP is largely occurring within hospital-based settings and in specialised clinics, which reflects patterns reported internationally [15, 24, 27, 53–56]. Unlike countries where pharmacists play an important role in community pharmacy [15, 19, 25, 52], only one study in this review explored community pharmacists’ views, reporting interest and perceived capability for independent prescribing, particularly for minor ailments [30]. However, as community pharmacists are primarily limited to supplying over-the-counter (OTC) medications, these findings can be interpreted as proposed future roles rather than descriptions of the existing legal framework. The included studies highlighted several facilitators, including specialised education and professional competence. An evaluation of Qatar University’s pharmacy curriculum confirmed that it effectively equips graduates with prescribing competencies, demonstrating a strategic educational effort to support the advancement of PP [42]. Interprofessional collaboration, institutional legislation, and supportive infrastructure emerged as critical facilitators and were clearly demonstrated in several regional initiatives. For instance, the establishment of a pharmacist-led diabetic clinic in Saudi Arabia [38] and the operation of an ambulatory clinical pharmacist-led multiple myeloma clinic in Qatar [46], where pharmacists are authorised to initiate or modify medication therapy through a CPA. These examples demonstrate the practical success of these facilitators, creating a pathway for PP across the region. The reported facilitators align closely with facilitators reported globally [19, 23–26, 54, 57].

Barriers to the implementation of PP were also substantial. The absence of national legislation emerged as a critical obstacle, creating inconsistencies in prescribing authority. Limited physician support was also reported as a significant barrier [30, 43]. Comparable challenges have been documented internationally, where regulatory uncertainty and professional resistance delayed the uptake of PP [21, 53–55]. The findings also revealed barriers such as limited laws and regulations, a lack of regulatory frameworks [29, 36, 42, 43], the absence of standardised definitions of PP models [45], and the limited availability of data, which confirms the effectiveness of PP [39]. Collectively, these findings highlight the importance of addressing barriers to support the safe and effective implementation of PP.

### Strengths and limitations

A key strength of this review is the comprehensive search strategy, which included a total of nine databases. Moreover, this review followed the recommendations in the PRISMA-ScR checklist to ensure transparency and consistency. However, there are limitations to consider, including inconsistent terminology regarding PP. Many studies employed broader descriptors, such as “pharmacist-led clinics” or “pharmacist interventions”, without specifying whether prescribing activities were involved. In some studies, the term ‘prescribing’ appeared to include the supply of OTC medications, which does not constitute PP. An additional limitation stems from the significant under-representation of studies from the majority of countries within the region. While this review has provided valuable insights into the four nations in which prescribing was addressed, the limited geographical scope means that the findings may not be generalisable to the entire region.

## Conclusion

This scoping review provides an overview of the current landscape of PP in Middle Eastern Arab countries. While the evidence remains sparse, findings suggest growing interest and limited early efforts, particularly in specialised clinical areas. Despite notable challenges, there is clear potential for PP to enhance clinical outcomes and healthcare efficiency. To translate this potential into practice, a rigorous effort is required to standardise the regulatory landscape, develop structured training and certification programmes, and foster a collaborative healthcare environment that embraces the pharmacist's evolving role.

## Supplementary Information

Below is the link to the electronic supplementary material.

Supplementary file1 (DOCX 15 kb)

Supplementary file2 (DOCX 66 kb)

## Data Availability

All data generated in this review are included in the manuscript and the supplementary materials.
