# Supplementary material for: Exploring the current status of pharmacist prescribing in Middle Eastern Arab countries: a scoping review
Source: Int J Clin Pharm. 2026 Mar 17;48(3):723–30. doi: 10.1007/s11096-026-02108-0 (PMC13175995; doi:10.1007/s11096-026-02108-0)
Supplement: Supplementary file 1 — Supplementary file1 (DOCX 15 kb) [file 11096_2026_2108_MOESM1_ESM.docx]

**Appendix 1. Medline search strategy**

| # | Searches |
| --- | --- |
| 1 | middle east/ or bahrain/ or iraq/ or jordan/ or kuwait/ or lebanon/ or oman/ or qatar/ or saudi arabia/ or syrian arab republic/ or united arab emirates/ or yemen/ |
| 2 | Bahrain/ |
| 3 | Iraq/ |
| 4 | Jordan/ |
| 5 | Kuwait/ |
| 6 | Lebanon/ |
| 7 | Oman/ |
| 8 | Palestine.mp. |
| 9 | Qatar/ |
| 10 | Saudi Arabia/ |
| 11 | Syria/ |
| 12 | United Arab Emirates/ |
| 13 | Yemen/ |
| 14 | Arab world/ or arab world.mp. |
| 15 | Arab/ or arab*.mp. |
| 16 | Gulf Cooperation Council.mp. |
| 17 | 1 or 2 or 3 or 4 or 5 or 6 or 7 or 8 or 9 or 10 or 11 or 12 or 13 or 14 or 15 or 16 |
| 18 | ''non-medical adj2 prescrib*''.mp. |
| 19 | ''independent adj2 prescrib*''.mp. |
| 20 | ''supplementary adj2 prescrib*''.mp. |
| 21 | ''collaborative adj2 prescrib*''.mp. |
| 22 | ''pharmacist adj2 prescrib*''.mp. |
| 23 | ''pharmacist-led adj2 clinic''.mp. |
| 24 | 18 or 19 or 20 or 21or 22 or 23 |
| 25 | 17 and 24 |
