# Supplementary material for: Exploring the current status of pharmacist prescribing in Middle Eastern Arab countries: a scoping review
Source: Int J Clin Pharm. 2026 Mar 17;48(3):723–30. doi: 10.1007/s11096-026-02108-0 (PMC13175995; doi:10.1007/s11096-026-02108-0)
Supplement: Supplementary file 2 — Supplementary file2 (DOCX 66 kb) [file 11096_2026_2108_MOESM2_ESM.docx]

| **Appendix 2. Findings of included studies related to pharmacist prescribing in Middle Eastern Arab countries** | | | | | | | | | |
| --- | --- | --- | --- | --- | --- | --- | --- | --- | --- |
| **Author (year)** | **Aim** | **Study design** | **Study setting** | **Country** | **Reported qualifications** | **Model of prescribing** | **Facilitators** | **Barriers** | **Study findings and recommendations** |
| Al-Otaibi et al. (2019) | To analyse pharmacist prescribing privileges in Saudi Arabia. | Cross-sectional study. A questionnaire was distributed electronically to 36 hospitals including pharmacy leaders, including directors of pharmacy, deputy directors, pharmacy quality managers, clinical pharmacy coordinators, or designated pharmacists representing the hospital’s director of pharmacy. | Hospital | Saudia Arabia | Not explored | -Total Parenteral Nutrition (TPN) with pharmacists acting as independent prescribers  -The therapeutic interchange system was introduced, allowing pharmacists to automatically substitute a patented drug with its generic equivalent of the same chemical name without requiring physician approval. | Not explored | Not explored | -Pharmacists in Saudi Arabia actively participated in prescribing and therapeutic interchange of medications under pharmacy law.  -Given that 32% of hospitals surveyed had pharmacist prescribing, it was recommended to increase the adoption of pharmacist prescribing across more hospitals in Saudi Arabia to optimise patient care.  -Pharmacists prescribed medications primarily through ambulatory care clinics, in which prescriptions were co-signed by a physician. Pharmacists could also prescribe medications under prescribing protocol, and they provided pharmacokinetic consultation.  -The majority of medications prescribed by pharmacists were over-the-counter (OTC) products and vitamins, followed by minerals and electrolytes used in TPN. Similarly, most medications prescribed through the therapeutic interchange programme included non-steroidal anti-inflammatory drugs (NSAIDs), vitamins, electrolytes used in TPN, and antihistamines. It was recommended to extend pharmacist prescribing to include a wider range of medications and other therapeutic agents, to improve patient outcomes.  The study found that the hospital's Computerised Physician Order Entry system was not configured to allow credentialed pharmacists to enter their own prescriptions, preventing them from exercising their prescribing authority electronically  It was recommended to improve and fully integrate this system to ensure better tracking and management of pharmacist-prescribed medications.  -The study highlighted the need for a fully implemented system that clearly defined pharmacists' authority in prescribing medications and conducting therapeutic interchange across Saudi Arabia. |

| **Appendix 2. (*cont’d*) Findings of included studies related to pharmacist prescribing in Middle Eastern Arab countries** | | | | | | | | | |
| --- | --- | --- | --- | --- | --- | --- | --- | --- | --- |
| **Author (year)** | **Aim** | **Study design** | **Study setting** | **Country** | **Reported qualifications** | **Model of prescribing** | **Facilitators** | **Barriers** | **Study findings and recommendations** |
| Alomi et al. (2019) | - To estimate economic outcomes and cost avoidance of pharmacist prescribing TPN to neonates, paediatrics and adults. | Economic modelling study. The study estimated cost avoidance associated with pharmacist-led prescribing and management of TPN services across 20 hospitals providing care for neonatal, paediatric, and adult patients. | Hospital | Saudi Arabia | Not explored | TPN with pharmacists acting as independent prescribers | Not explored | Not explored | -The study highlighted significant cost savings associated with pharmacist prescribing of TPN orders in Saudi Arabia. A data simulation from 20 hospitals demonstrated that the highest cost avoidance was observed in neonatal patients, followed by adult and paediatric populations.  -The study highlighted the need for further research to compare cost savings between pharmacists and physicians in TPN prescribing.  -Expanding pharmacists' roles in nutrition support services, particularly in prescribing TPN, aligned with the goals of Saudi Vision 2030 and the Ministry of Health (MOH) strategic plan and was strongly recommended for all healthcare institutions in Saudi Arabia. |
| Ajabnoor & Cooper (2020) | - To provide evidence of the current practice of pharmacists’ prescribing in Saudi Arabia  - To explore pharmacists’ perspectives for further extending their role as prescribers. | Cross-sectional study. A questionnaire was distributed electronically to 137 registered hospital pharmacists in Saudi Arabia. | Hospital | Saudi Arabia | Prescribers were more likely to have a PharmD degree,  completed residency  training, and practice in clinical pharmacy settings | -Collaborative prescribing (most common)  -Independent prescribing | The presence of institutional legislation supporting the adoption of prescribing in hospitals. | -Lack of appropriate training.  -Lack of national legislation to support pharmacist prescribing.  -Lack of time for pharmacists to take additional workload.  -Healthcare practice culture and limited support from physicians. | -Implication was that national legislation would standardise requirements needed for pharmacists to undertake prescribing. This could be in the form of completion of prescribing training in which pharmacists would be required to achieve a certain level of prescribing competency and pass tests to enable them to act as prescribers.  -There was support from hospital pharmacists for pharmacists’ prescribing.  -There was a positive attitude toward introducing legislation to support pharmacist prescribing nationally in Saudi Arabia.  -It was implied that hospitals should adopt national prescribing arrangements rather than institutional ones. |
| Alsuwayni & Alhossan (2020) | To evaluate health outcomes in diabetic patients managed in a pharmacist-led diabetes clinic, including HbA1C levels, routine screenings, medication adherence, and biomarkers associated with comorbid conditions. | Prospective cohort study. The study included 35 adult diabetic patients who referred to the pharmacist-led clinic and had at least three-month follow-up visits. | Pharmacist-led diabetes clinic in an academic hospital | Saudi Arabia | A board-certified ambulatory care clinical pharmacist and a certified diabetic educator. | Collaborative practice agreement (CPA) | -CPA facilitated flexibility and interdisciplinary collaboration.  -CPA positively impacted patient outcomes across settings and disease states.  -Ensuring prescribed medication quantities aligned with follow-up intervals and monitoring adherence facilitated better medication management. | -Discrepancy between prescribed medication quantities and scheduled follow-up intervals contributed to medication non-adherence.  -Discrepancies in prescription quantities and follow-up intervals led to reliance on refill clinics or delayed medication use. | -Establish clear regulatory guidelines and legislation to support pharmacist prescribing practices, ensuring safety, responsibility, and appropriate scope of practice.  -Provide structured training and certification programmes to prepare pharmacists for prescribing roles. This includes clinical training and assessment of competencies.  -Promote collaborative practice models where pharmacists work alongside physicians and other healthcare professionals, which can enhance acceptance and integration of pharmacist prescribing.  -Conduct pilot studies and further research to evaluate the outcomes, feasibility, and safety of pharmacist prescribing in the Saudi healthcare system.  -Raise awareness among stakeholders (healthcare providers, policymakers, patients) about the benefits and potential of pharmacist prescribing to gain broader support. |
| Alomi et al. (2020) | To introduce pharmacist prescribing as a new initiative in Saudi Arabia | The study was designed as a new initiative project. It followed a structured framework influenced by international business models, pharmacy project guidelines, and project management institution standards. The study included several key phases. |  | Saudi Arabia | Not explored | -Collaborative prescribing (most common).  - Independent prescribing (TPN prescribing only). | -Reduction in physician workload.  -Suitable expertise for prescribing, particularly among clinical pharmacists with PharmD training. | -Limited pharmacy regulations and laws, both locally and internationally, could hinder the implementation of pharmacist prescribing  -Absence of a strategic plan. The prescribing initiative might have faced challenges if the pharmacy lacked a strategic implementation plan.  - Absence of an administration planner can pose a threat to implementation of pharmacist prescribing. | -The pharmacist could prescribe medications through ambulatory care services, inpatient services, critical care services, oncology services and community pharmacy services.  -The pharmacist could prescribe various medications like OTC, TPN, cardiovascular medications, hormonal contraceptives and oncology medications.  -Clinical pharmacists' skills could be effectively utilised to provide additional services and improve patients' quality of life. |
| Abdallah et al. (2020) | To evaluate the Bachelor of Science in Pharmacy (BSc (Pharm) curriculum at Qatar University College of Pharmacy (QU CPH), for addressing international prescribing competencies, and to identify gaps related to these competencies in the curriculum. | Curriculum mapping. The Australian National Prescribing Service (NPS MedicineWise) Prescribing Competencies Framework which outlines seven key prescribing competencies, was used to map the BSc Pharm programme. Learning outcomes (LOs) from 62 courses were assessed for alignment with this framework. Identified LOs were then mapped to the 2017 Association of Faculties of Pharmacy of Canada educational outcomes, which underpin the BSc Pharm programme at (QU CPH). | Qatar University College of Pharmacy | Qatar | Not explored | CPA grants pharmacists a certain level of prescribing authority in specific hospital settings, such as anticoagulation and heart failure clinics. | -The BSc (Pharm) curriculum at QU CPH addressed most of the prescribing competencies listed in the Australian National Prescribing Services (NPS MedicineWise) Prescribing Competencies Framework. | -Gaps in the curriculum.  -Legislative gaps in respect of accessing patient information. | -Pharmacist prescribing has existed in Qatar as a CPA in some multidisciplinary care settings, e.g. anticoagulation clinics. However, it still very limited as it lack nationwide legal authority for pharmacists to prescribe medications.  - Policies related to prescribing may still be in the development stage, limiting students' access and exposure to these policies during their experiential learning.  -Certain competencies, including electronic prescribing and prescribing-related policies, were insufficiently covered or entirely absent in the curriculum.  -Mapping the curriculum plays a crucial role in shaping pharmacy graduates' prescribing practice expectations and guiding the development of future prescribing curricula. |
| Jebara et al. (2020a) | To determine the extent of agreement between key stakeholders in Qatar regarding a framework for the potential development and implementation of pharmacist prescribing | Quantitative, consensus-based, modified-Delphi study involving 33 key health-related stakeholders in Qatar. This study followed a modified Delphi approach, as the questionnaire was informed by a literature review and previous qualitative interviews. | Major healthcare institutions in Qatar (Ministry of Public Health, primary, secondary, and tertiary healthcare settings, and all health academic institutions). | Qatar | Pharmacist prescribers are required to practice within their area of competence and adhere to a governance framework aligned with the standards set by the Qatar Council for Healthcare Professionals (QCHP). | Collaborative prescribing model | Not explored | Not explored | -Consensus from key decision makers on a framework to support the development and implementation of pharmacist prescribing.  -Consensus was achieved for pharmacists prescribing within defined scopes, excluding controlled drugs because of legislation in Qatar which mandates that controlled drugs can only be prescribed by a licensed physician.  -Consensus around the need for targeted education and training of pharmacist prescribers to ensure safe and effective prescribing.  -It was agreed that pharmacist prescribers must complete a specific, accredited education and training programme before engaging in prescribing activities. Education programmes should align with international standards such as the UK independent prescribing model, combining university-based academic education with practical, hands-on training in a clinical setting.  - There was strong consensus regarding the governance of prescribing at the prescriber level, emphasising evidence-based practices and robust documentation to ensure high-quality prescribing. |
| Jebara et al. (2020b) | To explore the views of key stakeholders in Qatar regarding the potential development and implementation  of pharmacist prescribing. | Qualitative, semi-structured interviews with 37 key stakeholders holding strategic roles in policy influence, including directors of medical, pharmacy, and nursing departments, health-related academics, patient safety and quality directors, and professional regulators. | Organisations  and institutions involved in the practice, education, regulation, and governance of pharmacists include the Ministry of Public Health, healthcare settings across primary, secondary, and tertiary levels, community pharmacies, and academic institutions offering programmes in medicine, nursing, pharmacy, and pharmacy technician education. | Qatar | Not explored | Not explored | -The professional experience of non-medical prescribers in healthcare.  -Adherence to evidence-based guidelines and treatment protocols.  -Peers’, medical practitioners’, and patients’ support.  -Additional training to pharmacists before granting prescribing authority.  -Raising awareness among the public and healthcare professionals about pharmacists' education and qualifications.  -Establishing collaborations with countries that have successfully implemented pharmacist prescribing.  -Strengthening pharmacists' confidence in taking on prescribing responsibilities.  -Developing comprehensive standards and structured processes for implementation.  -Engaging a diverse range of stakeholders in the implementation of pharmacist prescribing. | -The absence of clearly defined roles for non-medical prescribers.  -The lack of dedicated time for prescribing activities and competing responsibilities.  -Limited confidence among some non-medical prescribers.  -Resistance from healthcare professionals and patients toward non-medical prescribers. | -Pharmacist prescribing has the potential for systematic development and integration within Qatar’s healthcare system  -Further research is needed to establish appropriate prescribing models and address key aspects such as education, training, and accreditation. |
| Alghadeer et al. (2021) | To assess glycaemic control by comparing the reduction in HbA1c between patients monitored in a pharmacist-led diabetic clinics vs. those monitored in physician-led diabetic clinics. | Retrospective observational study with a 12-month follow-up assessing glycaemic control in 52 diabetes clinic patients—24 managed in pharmacist-led clinics and 28 in physician-led clinics. Outcomes included changes in haemoglobin A1c (HbA1c), fasting blood glucose, blood pressure, and lipid profiles. | Diabetes management clinics at King Saud  University Medical City (KSUMC) | Saudi Arabia | Clinical pharmacists  who have sufficient training and/or a certain certificate for diabetes management | CPA | -Pharmacists’ professional experience and clinical knowledge.  -Institutional support and policy changes such as the establishment of the CPA. | -Reluctance to establish pharmacist-led clinics.  -The absence of local data and evidence that demonstrate the effectiveness of pharmacist care models.  -Limited provision of pharmacist-led clinics. | -This study showed a successful and comparable level of care that was not limited to achieving the patient’s targeted HbA1c, but extended to assessing other diabetes-related risks, and implementation of the guidelines’ recommended preventive measures.  -The pharmacist-led clinic changed the doses of diabetes medication for their patients more frequently compared to the physician-led clinic  -The pharmacist-led clinic had slightly better outcome management in terms of diet and exercise, blood sugar testing, foot care assistances, and empowering patient self-management, which are recommended to improve diabetes and prevent or minimise the chances of developing diabetes complications.  The integration of clinical pharmacists in diabetes and/or other chronic disease management within a collaborative practice agreement will enhance medication utilisation, improve disease-related outcomes, and correspondingly reduce cost and promote the overall patient experience. |
| Alnais & Altebainawi (2021) | To describe the history, establishment, structure, operations, and potential of Pharmacist-Led Diabetic Clinics (PLDCs) in delivering patient-centred care and to provide a framework for other MOH hospitals seeking to establish their own PLDCs. | Description of the establishment, structure and operations of a PLDC. | Hospital | Saudi Arabia | Clinical pharmacists with clinical privileges approved by the hospital, according to the guidelines of the Saudi Central Board for Accreditation of Healthcare Institutes (CBAHI). | CPA | -Institutional & Regulatory Support.  -Alignment of pharmacist prescribing roles with national health strategies (Saudi Vision 2030).  -Specialised pharmacy education such as PharmD and residency programmes.  -The establishment of CPA that clearly defines pharmacist prescribers ' role, responsibilities and services.  -Effective multidisciplinary team collaboration with physicians and other healthcare providers.  -Availability of clinic infrastructure and resources (e.g., counselling units, labs, patient records).  -Access to the electronic health records.  -Evidence of positive outcomes particularly in chronic disease management. | -Resistance of hospital physicians and administrators to the PDLC.  -Large numbers of patients and long waiting lists.  -Limited numbers of clinical pharmacists trained in diabetes management. | -This study reports the establishment of the first PLDC within MOH hospitals, the largest healthcare sector in Saudi Arabia, serving as a model for replication in other institutions.  -Clinical pharmacists across Saudi Arabia are encouraged to implement similar PLDCs to enhance therapeutic care for patients with diabetes.  -Future clinics should focus on increasing awareness among policymakers and hospital communities while expanding multidisciplinary involvement to address the challenges posed by a high patient volume. |
| Saadah et al. (2021) | To evaluate the outcomes associated with maximising the acceptance of clinical pharmacy interventions (CPI) using artificial neural networks (ANN), to determine whether pharmacists qualify for full independent prescribing authority, and to assess if accepting more CPI would improve healthcare outcomes such as length of hospital and intensive care stay, reduce readmissions, improve survival, or reduce costs | Non-interventional retrospective study conducted over eight months. The examined documented CPIs including 542 patients, 574 admissions, and 1694 CPIs. | Tertiary- and acute-care private  hospital | Jordan. | Clinical pharmacy team consisted of three clinical pharmacists with 4, 7, and 8 years of experience respectively, holding Doctor of Pharmacy or Master of Clinical Pharmacy certificates | Proposed model: independent prescribing | -Clinical pharmacists’ experience and expertise.  - Evidence of positive impact of CPI including a reduction in the length of hospital stay and potential cost savings. | -Physician rejection/ resistance to pharmacist prescribing.  -Challenges in demonstrating the benefits of a CPI when physicians decline it.  -Disagreement on pharmacotherapy decisions between pharmacists and physicians (20% disagreement rate). | -Clinical pharmacists in practice may become eligible for enhanced privileges, including the opportunity to advance to fully independent prescribing.  -There is a need for further evidence, of the effect of rejecting CPI, on secondary outcomes like intensive care units stay and mortality. |
| Stewart et al. (2021) | -To explore pharmacists’ aspirations and readiness  to implement pharmacist prescribing. | Mixed-methods design, cross-sectional survey and focus groups. The survey included 348 pharmacists, while 100 participated in the focus group. | The national health service (Hamad Medical Corporation, HMC) | Qatar | Not explored | Proposed model:  collaborative, supplementary, and independent model | -Access to medical records.  -Organisational and management support.  -The practice environment. | -Physician resistance.  -Current legislation and scope of practice. | -Key facilitators and barriers identified can be used in planning the implementation of pharmacist prescribing in Qatar and other countries at a similar stage of development.  -HMC pharmacists largely aspired to, and considered themselves ready, to be pharmacist prescribers with inpatient and outpatient settings most prepared for implementation.  -The majority considered themselves ready to undertake prescribing, particularly those in senior positions and classifying themselves innovators or early adopters.  -The highest levels of agreement, as measured through a questionnaire, were in relation to pharmacist prescribing being important for improving the safe use of medicines, the economic use of medicines and patient care outcomes, according to pharmacists at HMC, with community pharmacy considered least ready.  -The survey highlighted a need for enhanced training in critical areas such as therapeutics, physical assessment skills, and clinical decision-making. Therefore, a clear recommendation is to develop and implement targeted training programmes to address these skills, supporting pharmacists in effectively managing their expanded responsibilities. |
| Sadeq et al. (2022) | -To evaluate and investigate the perception and attitudes of community pharmacists toward independent prescribing and their perceived ability to diagnose and manage common health problems | Cross sectional design. A total of 220 licensed pharmacists completed a questionnaire consisting of five sections: demographic, confidence levels, perceived requirements and barriers for the community pharmacist independent prescribing process, and perceived competence in managing common medical conditions. | Community pharmacies in Al-Ain City, United Arab Emirates | United Arab Emirates | Not explored | Proposed model: independent prescribing by community pharmacists | - Access to patients’ medical records.  - Properly designed training programmes for pharmacists to improve their competencies.  - Regulations to provide permission for practising independent prescribing.  -Consideration of reimbursement. | - Patients’ and public acceptance of this service.  - Physicians' acceptance.  -Infrastructure, resources, staffing and financial support.  - Pharmacists’ knowledge and skills. | -A majority of community pharmacists (75.0%) reported feeling competent in managing minor ailments such as acute back pain and acne. In contrast, they expressed lower levels of perceived competence in the management of chronic conditions, including hypertension and asthma  -While 70.0% of pharmacists expressed confidence in their ability to engage in community pharmacist independent prescribing, only 58.0% reported confidence in their current knowledge and skills necessary for its implementation.  -Piloting and implementing community pharmacist independent prescribing services should be considered especially for minor ailments such as acute back pain, acne and allergic conjunctivitis.  -Additional training and education should be provided to enhance pharmacists’ confidence and competence in independent prescribing.  - Enhance training and education for community pharmacists to improve their knowledge and skills for prescribing, particularly in managing chronic diseases.  - Ensure community pharmacies are equipped with well-prepared consultation areas.  - Establish clear policies and guidelines, enabling safer and more informed clinical decision-making.  - Implement systems that provide pharmacists with necessary access to patient medical records.  - Develop strategies to improve collaboration and acceptance among physicians. |
| Almuqbil et al. (2023) | -To evaluate the quality of medical documentation  in anticoagulation clinics and investigate the differences  between pharmacist-led and physician-led clinic notes. | Retrospective cross-sectional study in which data were extracted from electronic health records of 160 patients. The patients were receiving anticoagulation therapy and being followed in two separate clinics. 160 patients were followed by the  physician-led clinic, and 171 patients were by the pharmacist-led clinic. Data from both clinics were included in the analysis. | Ambulatory care settings in  tertiary care hospital (King Khalid University Hospital) | Saudi Arabia | Clinical pharmacists | CPA | Not explored | Not explored | -Documentation was seen as a vital component in the healthcare. Standardised documentation can enhance reporting quality and improve patient care.  -Pharmacists demonstrated strong documentation practices compared to physicians, particularly in recording drug–drug and drug–food interactions and adhering to follow-up protocols.  -Pharmacists were found to follow the anticoagulation clinic protocol in terms of follow-up more than physicians. |
| Alshaiban et al. (2023) | -To evaluate the outcomes  of clinical pharmacists’ intervention in the control of warfarin International Normalised Ratio (INR) level and management  of warfarin therapy risk of bleeding and hospitalisation | Observational retrospective cohort study on 96 patients taking warfarin therapy in a clinical pharmacist-led anticoagulation clinic. | Tertiary care hospital (Prince Faisal Bin Khalid Cardiac Centre) | Saudi Arabia | Reported as trained, certified clinical pharmacist | CPA | -Availability of clinic infrastructure and resources.  -Access to the hospital information system.  -The establishment of pharmacist-led specialised clinics and protocol for prescribing practices.  -Collaboration between clinical pharmacists and physicians.  -Training, certification, and prescribing competency of clinical pharmacists.  -Comprehensive pharmacist interventions.  -Improved therapeutic outcomes.  -Enhanced patient compliance and follow-up.  -Acceptance by other healthcare providers. | -Limited physician access which could hinder collaborative prescribing.  -Lack of organisation and strategic planning for structured prescribing.  -Lack of scheduled appointments.  -Lack of patient attendance at scheduled follow-up appointments. | -The study highlighted the positive impact of clinical pharmacists' interventions on warfarin therapy outcomes.  -Their involvement improved target INR control, thereby reducing adverse drug reactions (ADRs) such as bleeding and preventing further complications, particularly in cardiac care.  -The findings emphasised that pharmacists’ contributions enhanced patients’ health-related quality of life during warfarin therapy.  -The study underscored the need for skilled pharmacy personnel in both routine patient care and critical care settings within primary care networks. |
| Ghasoub et al. (2024) | To describe the implementation process of an ambulatory clinical pharmacist (CP)-led multiple myeloma (MM) clinic, and to state the key activities performed by CPs in the clinic and assess the impact of this clinic on patient outcomes. | Retrospective data collection and analysis comparing patients treated before and after clinic implementation.  Patients were categorised as pre-clinic (treated before the clinic implementation and  postclinic group (patients managed in clinic after its implementation). | Pharmacist-led clinics in a national centre for cancer care and research. | Qatar | Not explored | CPA | -Constant changes in healthcare systems and continuous development in the pharmacy profession.  -More efficient use of physician time to focus on treating new cases.  -Timely management of drug-related problems, reported adverse drug reactions, improved treatment outcomes, and reduced medication errors. | -The lack of patient satisfaction assessment and cost-effectiveness evaluation limited the ability to demonstrate the implemented service’s value, effectiveness, and impact on patient outcomes. | -The clinical pharmacist-led multiple myeloma clinic in the ambulatory setting in Qatar effectively contributed to clinical care optimisation by providing timely and comprehensive support.  -Pharmacists' involvement in many aspects of cancer care, such as medication management, dose adjustments, and patient education, underlined their clinical capabilities and competence, supporting the extension of their role to include prescribing medications.  -The collaborative prescribing model used showed potential for application in other cancer care settings to improve patient safety and treatment effectiveness.  -The involvement of clinical pharmacists in patient care was well-supported by a 100% acceptance rate of their recommendations by physicians, underscoring the significant role they play in optimising patient outcomes. |
